# Mild phenotype in Molybdenum cofactor deficiency: A new patient and review of the literature

**DOI:** 10.1002/mgg3.657

**Published:** 2019-03-21

**Authors:** Barbara Scelsa, Serena Gasperini, Andrea Righini, Maria Iascone, Valeria G. Brazzoduro, Pierangelo Veggiotti

**Affiliations:** ^1^ Pediatric Neurology Unit V. Buzzi Children's Hospital Milan Italy; ^2^ Pediatric Rare Diseases Unit, Department of Pediatrics MBBM Foundation, ATS Monza e Brianza Monza Italy; ^3^ Department of Pediatric Radiology and Neuroradiology V. Buzzi Children's Hospital Milan Italy; ^4^ Laboratory of Genetic Medicine ASST Papa Giovanni XXIII Bergamo Italy

**Keywords:** *MOCS2*, molybdenum cofactor, Molybdenum cofactor deficiency, neurodevelopmental outcome, sulfite oxidase

## Abstract

**Background:**

Molybdenum cofactor deficiency (MoCD) is a rare autosomal‐recessive disorder that results in the combined deficiency of molybdenum‐dependent enzymes. Four different genes are involved in Molybdenum cofactor biosynthesis: *MOCS1, MOCS2, MOCS3,* and *GEPH*. The classical form manifests in the neonatal period with severe encephalopathy, including intractable seizures, MRI changes that resemble hypoxic‐ischemic injury, microcephaly, and early death. To date, an atypical phenotype with late‐onset has been reported in the literature in 13 patients.

**Methods:**

We describe a late‐onset and a relatively mild phenotype in a patient with *MOCS2 *homozygous mutation.

**Results:**

Pyramidal and extrapyramidal signs are recognized in those patients, often exacerbated by intercurrent illness. Expressive language is usually compromised. Neurological deterioration is possible even in adulthood, probably due to accumulation of sulfite with time.

**Conclusion:**

Sulfite inhibition of mitochondrial metabolism could be responsible for the ischemic lesions described in patients with MoCD or alternatively could predispose the brain to suffer an ischemic damage through the action of other insults, for instance intercurrent illness. It is possible that sulfite accumulation together with other external triggers, can lead to neurological deterioration even in adulthood. The role of other factors involved in clinical expression should be investigated to establish the reason for phenotypic variability in patients with the same mutation.

## 1 INTRODUCTION

Molybdenum cofactor deficiency (MoCD) is a rare autosomal‐recessive disorder that results in the combined deficiency of molybdenum‐dependent enzymes, including xanthine oxidase, sulfite oxidase, and aldehyde oxidase (Johnson et al., 2001). Four different genes are involved in Molybdenum cofactor biosynthesis: *MOCS1* (OMIM*603707), *MOCS2* (OMIM*603708), *MOCS3* (OMIM*609277), and *GEPH* (OMIM*603930) (Leimkühler, Freuer, Araujo, Rajagopalan, & Mendel, 2003). *MOCS1* mutations are responsible for more than half of the patients, followed by *MOCS2* (Mechler, Mountford, Hoffmann, & Ries, 2015). *GEPH*mutations have been described in two families, respectively, of Danish and of Algerian origin (Reiss et al., 2001,2011). Only one patient has been reported so far with *MOCS3* gene mutation (Huijmans et al., 2017).

The classical form manifests in the neonatal period with severe encephalopathy, including intractable seizures, MRI changes that resemble hypoxic‐ischemic injury, microcephaly, and early death (Mechler et al., 2015). In the literature, an atypical phenotype with late‐onset has been recognized and to date only 13 patients are reported (Alkufri et al., 2013; Arenas et al., 2009; Graf, Oleinik, Jack, Weiss, & Johnson, 1998; Hughes, Fairbanks, Simmonds, & Robinson, 1998; Huijmans et al., 2017; Johnson et al., 2001; Johnson, Wuebbens, Mandell, & Shih, 1988; Mayr et al., 2018; Megahed et al., 2016; Mize, Johnson, & Rajagopalan, 1995; Shih et al., 1977; Vijayakumar et al., 2011; Zaki et al., 2016).

In late‐onset MoCD patients, the onset is within the first 2 years of life. Usually the clinical manifestations include developmental delay, lens dislocation, extrapyramidal, and pyramidal symptoms often arising abruptly after an intercurrent illness. Seizures are less common comparing to the classical form (Zaki et al., 2016). The clinical course is variable including patients with predominant extrapyramidal signs, occurring even in adulthood, and patients with catastrophic neurological deterioration. Basal ganglia and dentate nuclei changes are often recognized as an isolated finding in MRI of patients with late‐onset and mild clinical course. On the other hand, MRI changes of patients with late‐onset and severe clinical course are similar to the early‐onset form of MoCD (diffuse brain atrophy, gliosis, arrested development of myelination, and cystic necrosis of cerebral white matter).

We describe a late‐onset and a relatively mild phenotype in a patient with *MOCS2 *homozygous mutation.

### 1.1 Ethical compliance

Informed consent for patient information to be published was provided by the parents of the child.

### 1.2 Case report

Our patient is the second child born to unrelated parents of Chinese origin. There is no family history of neurological or metabolic disorders in the family. She was born at term after an uneventful pregnancy and delivery. Her psychomotor development in the first 15 months of life was described as normal. The first symptoms of the disease were recognized after a febrile illness at age 16 months. She developed left side hemiparesis and was admitted to a hospital in China. Brain MRI performed in China was not available. The family had with them only a translation of the clinical report. Abnormal signal intensity was reported in the dentate nuclei and in the globus pallidi, predominantly in the right side (consistent with the side of the hemiparesis). The child returned to Italy after 4 weeks and was admitted to a local hospital. A second MRI was performed and a cystic cavitation of the globus pallidus was detected in the right side. She underwent a thrombophilic screening including protein C activity, protein S activity, antithrombin, lupus anticoagulant, lipoprotein A, homocysteine, PT, PTT, and antiphospholipid antibodies. A vascular stroke was suspected on the basis of the MRI and the increased level of lipoprotein A. She was given acetylsalicylic acid 50 mg per day to prevent further vascular strokes.

**Figure 1:**
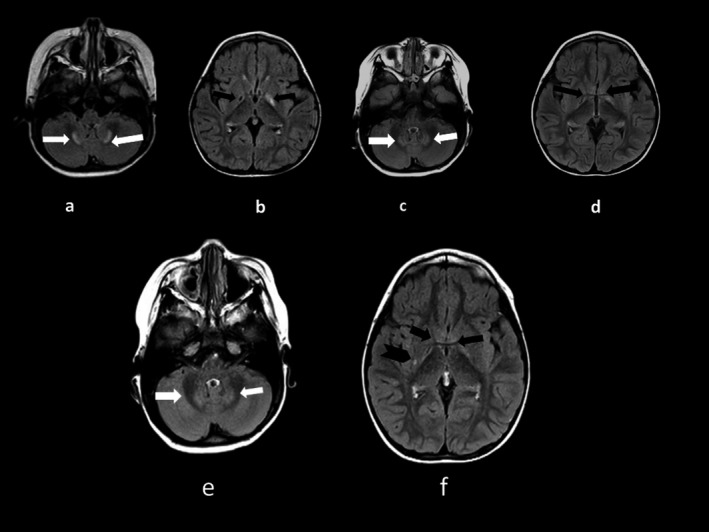
(a, b) T2‐FLAIR axial sections showing in the pallidus regions low signal intensity (cystic cavitation) in the right side and high signal intensity in the left side (black arrows); abnormal hyperintense signal in the dentate nuclei (white arrows) at 20 months. (c, d) corresponding sections, study confirming previous findings at 38 months. (e, f) T2‐FLAIR axial sections showing the same lesion of previous studies (white arrows), with an additional focal hyperintense lesion within the posterior striatum on right side at 3 years and 11 months (arrowhead)

The child came to our attention for the first time at 20 months. At admittance she was found to have a mild left hemiparesis, not impairing her ability to walk. She had normal behavior and social skills. She did not show any dysmorphic features and her head circumference was normal for age. She had an isolated myofibroma in her left wrist. Several investigations were carried out including coagulation profile, plasma and urinary amino acid, urinary organic acid, brain MRI, and EEG. MRI revealed areas of altered signal in the dentate nucleus and globus pallidus, bilaterally, with cystic cavitation on the right side (Figure 1a,b). EEG showed a sharp delta activity increased by sleep over the right parieto‐temporal area. Lipoprotein A was normal as well as the coagulation profile. Sulfite test was positive, S‐Sulfocysteine was found in urine, and plasma uric acid was low (Table 1). Initial genetic investigations by array‐CGH gave normal results. She was suspected to have a disorder of sulfur amino acids and sent to a referring center for metabolic disorders. Acetylsalicylic acid was discontinued. The child was submitted to further genetic investigation by trio‐based whole exome sequencing analysis. The patient is homozygous for a single base substitution of valine in place of phenylalanine (p.Val7Phe, NM_176806.3:c.19G>T) in *MOCS2* gene while both parents resulted heterozygous carriers. The variant was confirmed by Sanger sequencing in child and both parents.

**Table 1:** Biochemical profile of the patient

	Age		
	24 months	38 months	5 years
Sulfite test	+	+/−	+/−
S‐Sulfocystine (U)	↑	↑	↑
Taurine (U)	3,292 µmol/L (630–1580)	1676 µmol/L (630–1580)	304 µmol/L (17–230)
Cystine (P)	12 µmol/L (37–77)	5 µmol/L (37–77)	11 µmol/L (37–77)
Cystine (U)	9 µmol/L (15–35)	2 µmol/L (15–35)	1 µmol/L (4–11)
Uric acid (P)	1.8 mg/dl (2.4–5.7)	0.2 mg/dl (2.4–5.7)	0.8 (2.4–5.7)

At 21 months, she had her first and only focal seizure. The EEG confirmed the previous findings. No additional seizures were reported in the following months.

At 2 years of age the Griffiths Scale developmental quotient was 81 (low‐average range).

At 3 years of age she was submitted to a routine control including brain MRI. Clinically, she could walk outdoor independently, even though she had left side hemiparesis predominant in the inferior limb. The patient was found to have expressive language delay, although her understanding was adequate for age.

At 3 years and 2 months of age, after an intercurrent illness, she was admitted to our hospital for the sudden onset of extrapyramidal signs. The child displayed facial grimaces and choreic movements of the limbs. Since the onset of new neurological signs, she was submitted to MRI that confirmed the previous findings (Figure 1c,d). Coagulation profile, including PT, PTT, D‐dimer, was in the normal range, except for a slight elevation of fibrinogen (451 mg/dl‐reference values 150–400). The child was started again with acetylsalicylic acid 50 mg per day. The extrapyramidal movements elapsed in a few days.

At 3 years and 11 months, an additional lesion was found in the posterior part of the right striatum (Figure 1e,f) on brain MRI performed as a routine control. It is likely that the extrapyramidal signs described above were secondary to this additional lesion, not detected in the previous MRI (Figure 1c,d). The last MRI was performed at age 6 years and no variations were found.

Ophthalmological evaluations were carried out every year with normal results, except for a mild hypermetropia (OO+2). Lens dislocation has not been detected yet.

At 4 years of age, the Griffiths scales developmental quotient was 88 (low‐average range).

Our patient is currently 6 years and 11 month old. She is seizure‐free and has left side hemiparesis predominant in the lower limb, not impairing her ability to walk. She is not following a specific diet, although she is still taking acetylsalicylic acid. She is attending mainstream school with support for language impairment.

At age 6 years and 11 months, the scores obtained with a nonverbal intelligence test (C‐TONI/ Comprehensive Test of Non Verbal Intelligence) were above average (IQ 114).

## 2 DISCUSSION

Two forms of MoCD are currently recognized. In the classical severe form of MoCD, the onset is in the first month of life with intractable seizures, feeding difficulties, quadriplegia, and early death (Johnson et al., 2001). Dysmorphic features are also described in most children with the classical form: frontal bossing, full cheeks, widely spaced eyes, elongated palpebral fissures, thick lips, and long philtrum (Parini et al., 1997). Lens dislocation is often reported but only in children surviving the neonatal period (Mechler et al., 2015).

A late‐onset form has also been reported in literature in 13 patients (Alkufri et al., 2013; Arenas et al., 2009; Graf et al., 1998; Hughes et al., 1998; Huijmans et al., 2017; Johnson et al., 2001, 1988; Mayr et al., 2018; Megahed et al., 2016; Mize et al., 1995; Shih et al., 1977; Vijayakumar et al., 2011; Zaki et al., 2016). Table 2 below compares the clinical, genetic, neurological, and neuroradiological findings of those with our patient. The clinical course of late‐onset MoCD includes patients with neurological severe deterioration and patients with milder manifestations. When the disease begins in the first year of life, the outcome is usually severe with deterioration of motor and intellectual skills, very similar to those surviving the classical form of MoCD. When the disease begins after the first year of life, patients have predominantly extrapyramidal and focal pyramidal signs often occurring and/or worsening in the contest of intercurrent illness (Table 2). Marfanoid habitus and mild dysmorphic features are described in few patients (Huijmans et al., 2017; Mayr et al., 2018; Mize et al., 1995; Zaki et al., 2016). Lens dislocation is generally detected after 4–5 years of age. Hughes and Alkufri describe a child who presented only lens dislocation in infancy. She developed parkinsonism and dystonia after age 23 years (Hughes et al., 1998 and Alkufri et al., 2013). All patients have some degree of language delay especially in the expressive ability. In most studies, cognitive functions are not described with formal tests, but mild intellectual disability is reported in the majority of patients. Seizures are not very frequent and controlled by antiepileptic medications. Our patient is very similar to those with mild clinical course reported in the literature (Arenas et al., 2009; Huijmans et al., 2017; Johnson et al., 2001,1988; Mayr et al., 2018; Mize et al., 1995; Shih et al., 1977; Vijayakumar case 2 et al., 2011). She was presented with pyramidal signs in the second year of life after a viral illness. Transient extrapyramidal signs were detected at 3 years of age after an intercurrent infection. The clinical course in our patient seems to be mild, but the child is currently only 6 years old.

**Table 2:** Review of patients described in the literature with mild phenotype of MoCD

	Shih et al. (1977)‐Johnson et al. (1988)	Mize et al. (1995)	Graf et al. (1998)	Hughes et al. (1998) and Alkufri et al. (2013)		Johnson et al. (2001)	Arenas et al. (2009)	Vijayakumar et al. (2011)		Zaki et al. (2016)	Megahed et al. (2016)	Huijmans et al. (2017)	Mayr et al. (2018)	Current study 2019
				Case 1	Case 2 (sister)			Case 1	Case 2					
Age at onset (months)	17	24	6	12	NA	15	24	8	24	6	1	14	7	16
Dysmorphic features	no	Yes (marfanoid habitus)	no	no	no	macrocephaly	NA	no	no	Yes (frontal bossing, depressed nasal bridge, anteverted nares, retrognathia, long philtrum, and low set ears.)		Yes (marfanoid habitus)	Yes (marfanoid habitus)	no
Psychomotor development	delay	delay	delay	delay	Normal, deterioration at 23 years of age	Delay	delay	delay	delay	delay	delay	Delay (IQ 50)	Delay	delay
Behavioral disorders	yes (intermittent)	no	no	Yes(irritability)	yes (in infancy mild attention deficit, in adulthood apathy)	no	yes (hyperkinesia)	no	no	no	yes	yes (autistic features)	yes (autistic features)	no
Extrapyramidal signs	yes	Yes (coordination deficit)	yes	yes	Yes (at 23 years of age)	Yes	no	yes	no	yes	no	no	Yes	Yes (intermittent)
Ophthalmological findings (age‐years)	Lens dislocation (4)	Lens dislocation (8)	Lens dislocation (2)	no	Lens dislocation (6)	no	Lens dislocation ‐myopia(4)	no	Lens dislocation (5)	no	no	strabismus	Hyperopia	no
Worsening with intercurrent illness	yes	yes	NA	yes	NA	yes	no	yes	no	yes	NA	no	Yes	yes
neuromotor and intellectual deterioration	Yes	No	yes	yes	Yes in adulthood	no	no	yes	no	yes	no	no	no	no
seizures	yes	no	no	yes	No	no	no	yes	no	yes	yes	no	no	Yes (one)
Pyramidal signs	yes (hemiplegia)	yes (hemiplegia)	yes	no	No	no	no	yes	no	yes	Yes (hemiplegia)	yes	no	yes
Independent walking	yes	yes	no	no	Unstable gait after 23 years of age	yes	yes	no	yes	no	Yes (unstable)	yes (unstable)	yes	yes
Feeding difficulties	no	No	NA	Yes	Yes after 23 years of age	No	no	yes	no	yes	no	no	No	no
Language development	episode of aphasia	Delay	NA	Regression after 17 months‐absent at last examination	Normal‐ regression after 23 years of age (severe dysphonia‐anarthria)	Delay (expressive language)	Delay (expressive and receptive language)	delay	Delay (verbal dyspraxia)	delay	Delay	Delay (expressive language)	Delay (expressive language)	Delay (expressive language)
Outcome at last examination (age‐y)	Mild‐moderate (4)	Moderate (22)	Severe (2)	Severe (3)	Severe (23)	Mild (4.5)	Mild (7)	Severe (4)	Mild‐Moderate (7)	Severe (death at 5.5 years)	Moderate (6)	Moderate (17)	Mild (14)	Mild (6)
Uric acid (plasma)	Normal	↓	↓	↓	↓	normal	normal	↓	normal	normal	NA	*N*‐↓	↓	↓
S‐sulfocysteine	↑	↑	↑	NA	NA	↑	↑	NA	NA	↑	↑	↑	↑	↑
Sulfi test	+	+	+	+	+	+	+	NP	+	NP	NA	‐	+	+
MRI	NP	LN	BG‐SWM	BG‐DN	BG	BG‐cortical dysplasia	DN‐parietal cleft	LN‐A	DN	BG‐SWM‐A	A	SWM	BG‐SWM	BG‐DN‐LN
treatment	Antiepileptic drugs	none	Antiepileptic drugs	Antiepileptic drugs	Levodopa prednisolone	none	no	Antiepileptic drugs	none	Antiepileptic drugs	Antiepileptic drugs, Omega3, B‐vitamins	Low‐methionine diet	Low‐methionine diet	Acetylsalicylic acid
Molecular analysis^a^	NP	NP	NP	*MOCS2 * heterozygous variants: NM_004531.4:c.377+1G>C; and NM_004531.4:c.539_540del, p.Lys180fs	*MOCS2 *heterozygous variants: NM_004531.4:c.377+1G>C; and NM_004531.4:c.539_540del; p.Lys180fs	*MOCS2*‐heterozygous variants: NM_176806.3:c.16C>T; p.Gln6ter and 19G>T and NM_176806.3:c.19G>T; p.Val7Phe	*MOCS1*‐homozygous variant: NM_001075098.3:c.^a^7+6T>C	*MOCS1*C>t (Arg91 Trp)	*MOCS1*‐homozygous variant: NM_001075098.3:c.^a^7+6T>C	*MOCS2 *homozygous variant: NM_176806.3: c.3G>A p.Met1?	*MOCS2 *homozygous variant NM_176806.3: c.3G>A p.Met1?	*MOCS3 *homozygous variant: NM_014484.4:c.769G>A, p.Ala257Thr	*MOCS1 *Homozygous c.1338delG	*MOCS2*‐ homozygous variant: NM_176806.3:c.19G>T; p.Val7Phe

Note

NA: not available; NP: not performed; N: normal; IQ: intelligence quotient; BG: basal ganglia lesions; DN: dentate nuclei lesions; LN: lentiform nuclei lesions; SWM: abnormality of subcortical white matter‐A: atrophy.

a Following HGVS nomenclature.

There is not clear genotype‐phenotype correlation in late‐onset MoCD. As it is shown in Table 2, *MOCS2* and *MOCS1* mutations are described in the majority of patients. *MOCS3*mutation was found in only one patient (Huijmans et al., 2017). Our patient displays the same V7F substitution described in the patient reported by Johnson, but in both alleles (Johnson et al., 2001). The mild phenotype of this child was attributable to the residual activity of the V7F allele (Johnson et al., 2001; Leimkuhler et al., 2003). It is possible that a residual activity of molybdenum cofactor enzymes is responsible for late‐onset and milder phenotypes in MoCD (Mayr et al., 2018). However, this is true only in part in fact the same mutation in the same family can be responsible for different clinical courses (Alkufri et al., 2013; Hughes et al., 1998; Vijayakumar et al., 2011). The phenotype is probably a spectrum of manifestations starting from neonatal period to adulthood. Even though the role of other factors involved in clinical expression is still unclear, intercurrent illness seems to act as a trigger of neurological manifestations.

The diagnosis is usually established through biochemical parameters. However, the biochemical findings can be misleading and elevated urinary excretion of S‐sulfocysteine seems to be a more constant finding. Some patients might have false negative sulfite test and normal plasma uric acid (Reiss et al., 2011). For that reason, the diagnosis can be missed especially in late‐presenters with mild symptoms.

Brain MRI can show some peculiar aspects resembling, in the most severe patients, hypoxic‐ischemic encephalopathy (Appignani, Kaye, & Wolpert, 1996). MRI changes include brain atrophy, polymicrogyria, pontocerebellar hypoplasia, and cystic necrosis of cerebral white matter (Vijayakumar et al., 2011). Basal ganglia and dentate nuclei changes are often recognized as an isolated finding in late‐presenters with mild clinical course, including our patient (Vijayakumar et al., 2011).

The mechanism of brain damage is likely related to sulfite toxicity (Zhang, Vincent, Halliwell, & Wong, 2004). As already stated, neuroradiological findings can mimic hypoxic‐ischemic encephalopathy (Appignani et al., 1996). In fact, both conditions share similar pathogenic mechanism of insult, even though the etiology is striking different. In MoCD, it is the sulfite accumulation that inhibits mitochondrial metabolism, in particular glutamate dehydrogenase. Glutamate toxicity could be responsible for the ischemic lesions described or alternatively could predispose the brain to suffer an ischemic damage through the action of other insults, for instance intercurrent illness (Zhang et al., 2004). It is likely that continuous sulfite accumulation on the basal ganglia is responsible for the neurological deterioration (Alkufri et al., 2013). Nevertheless, the activation of the inflammatory cascade could also be responsible for additional brain damage and exacerbation of neurological symptoms. Our patient showed a slight elevation of fibrinogen in the contest of disease exacerbation. This elevation might be interpreted as an indirect sign of inflammatory cascade activation. In our patient, resolution of extrapyramidal signs occurred after acetylsalicylate was reintroduced. However, the role of salicylate is uncertain in stabilizing this condition and cannot be recommended. Even though the evidence for this secondary mechanism of injury has not been established, this observation may lead to additional treatment options especially for late MoCD presenters. However, the administration of steroid in the patient described by Alkufri gave limited improvement of parkinsonism and dystonia (Alkufri et al., 2013). With this regard, Kumar suggested that NMDA‐R antagonists, calcium influx, and calpain activity blockers could prevent neurotoxicity in a MoCD murine model (Kumar et al., 2017).

In the attempt to reduce sulfite accumulation, several treatments have been suggested with limited benefit. Cyclic pyranopterin monophosphate (cPMP) is the only effective treatment for *MOCS1* patients, even though cannot reverse brain injuries already established before treatment (Lubout et al., 2018).

No treatment is currently available for patients with *MOCS2* mutations, but pyridoxine supplementation seems to relieve some of the symptoms.

## 3 CONCLUSION

In patients with mild unexplained neurological symptoms, we suggest to consider MoCD as differential diagnosis by performing uric acid, sulfitest, and urinary amino acids. Patients with mild MoCD should be followed up until adult life. It is possible that sulfite accumulates with time and together with other external triggers, can lead to neurological deterioration even in adulthood. The role of other factors involved in clinical expression should be investigated to establish the reason for phenotypic variability in patients with the same mutation. The evidence that ischemic damage could also be induced by inflammatory stimuli would open the way to neuroprotection treatments with possible improvement of the outcome, at least in some patients.

## CONFLICT OF INTEREST

The Authors declare that there is no conflict of interest.
